# Long-term retention of non-funcionalized carbon nanotubes in nod mice and its influence on the evolution of autoimmune diabetes

**DOI:** 10.1186/1758-5996-7-S1-A209

**Published:** 2015-11-11

**Authors:** Daniela da Silva Camilo, Adriel dos Santos Moraes, Fernando Pradella, Guliherme Antonio Dutra Moraes, Rosemeire Florença de Oliveira de Paula, Elaine Conceição de Oliveira, Gustavo Ferreira Simões, Alexandre Leite Rodrigues de Oliveira, Helder José Ceragioli, Vitor Baranauskas, Aureo Tatsumi Yamada, Alessandro Santos Farias, Leonilda Maria Barbosa dos Santos, Walkyria Mara Gonçalves Volpini

**Affiliations:** 1 UNICAMP, Campinas, Brazil

## Background

The perspectives of using carbon nanotubes (CNTs) in medicine evoked many researches aiming to evaluate their risks on the human health as well as on the environment.

## Objective

This study focuses the long-term effects of the systemic administration of non-functionalized multi-walled carbon nanotubes (MWCTNs) on the evolution of spontaneous autoimmune diabetes in Non-Obese Diabetic (NOD) mice.

## Materials and methods

The protocol consisted in treating 6 weeks old NOD/Uni mice with a single intra-peritoneal dose of MWCTN (100 μg/animal) or the vehicle Pluronic (control group). Mice were followed during 24 weeks.

## Results

Histological data confirmed that the non-functionalized MWCTN had been absorbed and kept retained into the phagocytes of the peri-pancreatic lymph nodes, spleen and liver, causing a granulomatous inflammatory response. Even though no differences were found in the frequency of the development of clinical diabetes or in the morphological characteristics of the pancreatic insulitis, female NOD mice treated with MWCTN presented a significant higher fluctuation of the average glycaemia compared to the control group during the entire study (p<0.0001; Wilcoxon), with a tendency to an abbreviation of clinical diabetes onset. Analyses of the pro-inflammatory and the anti-inflammatory responses in the peri-pancreatic lymph nodes revealed the induction of a Th1 response in the treated animals, between 8 and 14 after MWCTN exposition, with an increase of the expression of IFNγ and a reduced expression of TGFβ. This profile remained until the end of the study, 24 weeks after MWCTN injection.

## Conclusion

These data show that MWCTN may be retained for at least 6 months in lymph nodes and pancreatic ducts of NOD mice. Therefore, despite the evident need of modifying nanotubes, it is important to consider the high risks of exposing these molecules to humans, since chronic inflammation may be related to the development and/or the aggravation of autoimmune diseases.

